# PolyCTLDesigner: a computational tool for constructing polyepitope T-cell antigens

**DOI:** 10.1186/1756-0500-6-407

**Published:** 2013-10-10

**Authors:** Denis V Antonets, Sergei I Bazhan

**Affiliations:** 1 State Research Center of Virology and Biotechnology “Vector”, Koltsovo, Novosibirsk Region, Russian Federation

**Keywords:** T-cell epitope, Polyepitope, Cytotoxic T cell, Transporters associated with antigen processing, Proteasome, Directed weighted graph, Travelling salesman problem

## Abstract

**Background:**

Construction of artificial polyepitope antigens is one of the most promising strategies for developing more efficient and safer vaccines evoking T-cell immune responses. Epitope rearrangements and utilization of certain spacer sequences have been proven to greatly influence the immunogenicity of polyepitope constructs. However, despite numerous efforts towards constructing and evaluating artificial polyepitope immunogens as well as despite numerous computational methods elaborated to date for predicting T-cell epitopes, peptides binding to TAP and for antigen processing prediction, only a few computational tools were currently developed for rational design of polyepitope antigens.

**Findings:**

Here we present a PolyCTLDesigner program that is intended for constructing polyepitope immunogens. Given a set of either known or predicted T-cell epitopes the program selects N-terminal flanking sequences for each epitope to optimize its binding to TAP (if necessary) and joins resulting oligopeptides into a polyepitope in a way providing efficient liberation of potential epitopes by proteasomal and/or immunoproteasomal processing. And it also tries to minimize the number of non-target junctional epitopes resulting from artificial juxtaposition of target epitopes within the polyepitope. For constructing polyepitopes, PolyCTLDesigner utilizes known amino acid patterns of TAP-binding and proteasomal/immunoproteasomal cleavage specificity together with genetic algorithm and graph theory approaches. The program was implemented using Python programming language and it can be used either interactively or through scripting, which allows users familiar with Python to create custom pipelines.

**Conclusions:**

The developed software realizes a rational approach to designing poly-CTL-epitope antigens and can be used to develop new candidate polyepitope vaccines. The current version of PolyCTLDesigner is integrated with our TEpredict program for predicting T-cell epitopes, and thus it can be used not only for constructing the polyepitope antigens based on preselected sets of T-cell epitopes, but also for predicting cytotoxic and helper T-cell epitopes within selected protein antigens. PolyCTLDesigner is freely available from the project’s web site: http://tepredict.sourceforge.net/PolyCTLDesigner.html.

## Findings

One of the most promising approaches to designing more efficient and safer vaccines is construction of artificial polyepitope antigens [1-6]. Their advantages over conventional vaccines include reduced risk of developing autoimmunity and other pathological conditions since such constructions doesn’t contain whole microbial molecular structures and epitopes, sharing profound similarity to human proteins. Besides, polyepitopes may contain both cytotoxic (CTL) and T-helper epitopes belonging to different antigens, including those of distinct pathogenic microorganisms, thus making possible to induce immune responses with a wider specificity. In addition, polyepitopes may be designed taking into account the prevalences of certain HLA class I molecules allomorphs within the target human population or even within an individual patient. Polyepitopes may also be constructed in a way maximizing efficiency of processing and presentation of the majority of included epitopes [4,7-9]. Additional signal sequences (for example, N-terminal ubiquitin, N-terminal leader peptide, and C-terminal fragment of human LAMP-1 protein) could be introduced into target polyepitopes to increase their efficiency of stimulating either CD8+ and/or CD4+ T-cell response [10-14]. However, despite numerous efforts towards constructing artificial polyepitope immunogens and evaluating their immunogenicity and protectivity [2,4-9,15,16] and despite numerous computational methods developed to date for predicting T-cell epitopes [17-20], proteasomal cleavage sites [21-25], and peptide binding to TAP (transporters associated with antigen processing) [24,26-28], only a few computational tools intended for rational design of polyepitope T-cell immunogens were developed to date [29,30].

Both interaction of peptides with MHC molecules and peptides binding to TAP complex are sufficiently specific for certain amino acid patterns. Proteasomal and immunoproteasomal cleavage sites are also to a considerable degree determined by the amino acid sequences of antigens and degenerate amino acid motifs that determine the efficiency of proteasomal cleavage sites are currently known [22,31] as well as the motifs determining the affinities of oligopeptides binding to TAP [26-28]. It has been shown that a concurrent prediction of peptide binding affinities for MHC molecules and TAP decreases false positive rate when predicting T-cell epitopes [26,27]. As it has been experimentally demonstrated, that introduction of spacer sequences to between individual epitopes could considerably increase the ability of such polyepitope constructs to induce cytotoxic T-cell immune responses [4,7,9]. Epitope rearrangements within the polyepitopic constructs were also found to significantly influence their immunogenicity and it was also hypothesized that while constructing the polyepitope antigen one should minimize the number of nontarget junctional epitopes [9].

It has been shown that longer peptides transported into the endoplasmic reticulum (ER) undergo N-terminal trimming by ER aminopeptidases (ERAPs) to allow them to bind MHC class I molecules [32], and that C-termini of the epitopes are preferentially generated by proteasomal cleavage [33-35]. Thus individual epitopes within a polyepitope should be arranged in a way providing sufficiently efficient proteasomal cleavage sites at their C-termini while, if necessary, their N-termini might be flanked with certain amino acid residues to optimize their binding to TAP.

The goal of this work was to develop a program intended for rational design of polyepitope T-cell antigens with a special focus on optimizing their immunogenicity via selecting amino acid spacer sequences for each pair of epitopes and choosing the optimal ordering of the epitopes within the polyepitope.

A program named PolyCTLDesigner was developed. Given a set of either known or predicted CTL epitopes, PolyCTLDesigner predicts affinity of their binding to TAP and then N-termini of inefficient binders are extended with certain flanking residues using the model created by Peters et al. [26], implying that the first three N-terminal amino acid residues of the peptide and the last C-terminal one are the major contributors to its binding to TAP.

Then all possible pairs of obtained oligopeptides are produced and PolyCTLDesigner predicts the proteasomal and/or immunoproteasomal cleavage sites using the models developed by Toes et al. [22]. According to the chosen model, a spacer motif containing up to six amino acid residues may be added when necessary after the C-terminal residue of an epitope in order to optimize the cleavage. For example, if the sequence ADLVKV is selected as a spacer, PolyCTLDesigner tests the following spacers: A, AD, ADL, ADLV, ADLVK, and ADLVKV and it additionally considers the variant of a direct junction of the epitopes. Besides, the program can also use degenerate motifs, such as [ARSP][DLIT][LGA][VKA]. In this case, all possible spacer sequences are generated and tested. An optimal spacer is determined for each pair of epitopes; it should (i) provide formation of the least number of non-target epitopes at the epitopes junction; (ii) form efficient proteasomal cleavage site at the C terminus of the first of the epitopes in the pair; and (iii) it should be the shortest of all, when the remaining parameters are equal. The optimal spacer sequence is selected according to the following ranking function:

$$\begin{array}{ll} W\left(\mathrm{pep}1,\;\mathrm{pep}2,\;\mathrm{ss}\right)= & \left(\sum_{\mathrm{HLA}}\mathrm{ran}k_{\mathrm{HLA}}\times \mathrm{fre}a_{\mathrm{HLA}}\right)\\ & +\mathrm{len}\left(\mathrm{ss}\right)+0.5\\ & \times \min\left(\mathrm{ran}k_{\mathrm{pr},}\;\mathrm{ran}k_{\mathrm{impr}}\right)\\ & +0.05\times \left(4-{\text{rank}}_{\mathrm{HLA}}\right)+0.05\\ & \times N_{\mathrm{eps}}\;+0.05\times N_{\mathrm{HLA}}+0.25\\ & \times \mathrm{ran}k_{\mathrm{pr}}+0.25\times \mathrm{ran}k_{\mathrm{impr}} \end{array}$$

where *W* is the weight (rank) of spacer sequence *ss* between the epitopes *pep1* and *pep2*; *rank*_*HLA*_ is the rank of non-target junctional epitope predicted to be the most efficient binder for HLA class I allele *HLA*; *freq*_*HLA*_ is the genotypic frequency of that allele within the population of interest (HLA alleles genotypic frequencies were taken from dbMHC [36]); *len(ss)* is the length of spacer *ss*; *rank*_*pr*_ corresponds to the rank of proteasomal cleavage site predicted at the *pep1* C-terminus (this value ranges from 1 to 11 with 1 and 11 corresponding to the most and the least efficient proteasomal cleavage, respectively); *rank*_*impr*_ is the rank of immunoproteasomal cleavage site; ${r\overset{¯}{an}k}_{\mathrm{HLA}}$ designates the mean *rank*_*HLA*_ value; *N*_*eps*_ is the number of predicted junctional epitopes and *N*_*HLA*_ is the number of HLA alleles predicted to bind non-target epitopes with sufficient affinity (currently PolyCTLDesigner predicts T-cell epitopes with our program TEpredict [37], that was recently updated); *rank*_*HLA*_ value of 1 corresponds to moderate binding affinity (6.3 ≤ pIC_50_ < 7.3), the value of 2 corresponds to high affinity (7.3 ≤ pIC_50_ < 8.3) and 3 corresponds to the highest affinity (with predicted pIC_50_ value ≥ 8.3). Thus the optimal spacer sequence should have the least weight.

**Figure 1:**
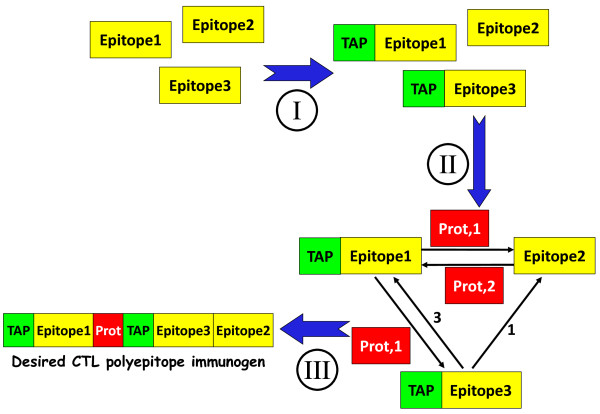
**PolyCTLDesigner workflow. (I)** Prediction of affinity of peptides binding to TAP and addition of up to 3 N-terminal flanking amino acid residues (when necessary); **(II)** selection of optimal spacer sequence for each peptide pair (the optimal spacer, selected by the ranking function for each peptide pair, should meet the following criteria: it should provide formation of proteasomal cleavage site at the C-terminus of the first peptide; it should provide the least number of non-target junctional epitopes; and it should have the shortest possible length) and construction of directed weighted graph with nodes corresponding to target epitopes and with edges corresponding to allowed variants of their combinations; and **(III)** construction of desired polyepitope immunogen amino acid sequence which is determined as the longest simple path with a minimal weight.

After optimal spacers are selected for each pair of epitopes, PolyCTLDesigner constructs an incomplete directed graph with nodes corresponding to peptides (epitopes) and edges corresponding to allowed epitope matchings. Each edge has two parameters: the optimal spacer sequence and its weight which was calculated by the ranking function described above. The constructed weighted digraph is in turn transformed into a complete one by adding edges corresponding to disallowed epitope matchings; their weights are set to 5000 while the weights of allowed epitope matchings usually don’t exceed 10. The sequence of desired polyepitope antigen can be determined as the least weighted complete simple path in the constructed weighted digraph, and as one can see this task is related to the travelling salesman problem (TSP). To find optimized sequence of polyepitope antigen PolyCTLDesigner uses either greedy nearest neighbor approach (only in the case of a nondegenerate spacer sequence), or genetic algorithm-based TSP-solver implemented in PyEvolve library [38]. The main steps of PolyCTLDesigner algorithm are shown in Figure 1.

Building the graph is the most time consuming step and for a set of 40–50 peptides it can take about 6–8 hours to be completed on typical desktop personal computer. However in future we plan to implement parallelized algorithm to reduce time consumption.

To illustrate the importance of poly-CTL-epitope optimization we performed a small theoretical analysis: six well-defined HLA-A*02:01-restricted HIV-1 CTL epitopes were used to produce artificial polyepitopes using our PolyCTLDesigner program. It was found that probability of selecting an optimal epitopes permutation at random was less than 0.00139 and only 17% of all possible polyepitope constructs did not contain inefficient proteasomal cleavage sites between target CTL epitopes. Besides, the choice of spacer sequences can have a great impact on proteasomal cleavage efficiency, especially for certain peptides. The detailed description of the study and its results can be found in Additional file 1.

In addition, PolyCTLDesigner is also able to assist in constructing polyepitope fragments containing T-helper epitopes. Currently T-helper epitopes can be predicted with ProPred models [39] (based on TEpitope models [40]). From a set of proposed antigens the program selects peptide fragments having 20–40 amino acid residues in length which contain the maximal number of overlapping T-helper epitopes restricted by maximal repertoire of HLA class II allomorphs. Then, each fragment is extended by five amino acid residues at both C- and N-terminals, since residues flanking the core epitope can play an important role in binding to T-cell receptors of CD4+ T lymphocytes [41]. The fragments containing T-helper epitopes can be joined through [KR][KR] motif, which form cleavage sites for several lysosomal cathepsins involved in antigen processing. It has been shown that such motifs can increase the immunogenicity of individual T-cell epitopes [42,43].

The developed software realizes a rational approach to designing highly immunogenic poly-CTL-epitope antigens and can be used to develop new candidate polyepitope vaccines. The current version of PolyCTLDesigner is integrated with our TEpredict program for predicting T-cell epitopes, and thus it can be used not only for constructing the polyepitope antigens based on preselected sets of T-cell epitopes, but also for predicting cytotoxic and helper T-cell epitopes within selected protein antigens. In addition, PolyCTLDesigner allows the user to select a minimal set of epitopes covering a specified repertoire of allelic variants of HLA molecules with a desired level of redundancy. The program was implemented using Python programming language and can be used either interactively or through scripting, which allows the users familiar with Python to create custom pipelines. PolyCTLDesigner source code can be found in Additional file 2 and it is also freely available at the project’s web site http://tepredict.sourceforge.net/PolyCTLDesigner.html.

## Methods

PolyCTLDesigner was implemented using the Python programming language. The affinity of peptides binding to TAP was predicted and the flanking sequences were selected using the model developed by Peters et al. [26]. The models by Toes et al. [22] were used to predict proteasomal and/or immunoproteasomal processing and to select spacer sequences to optimize polyepitope processing. The procedures involving graphs were realized using Python graph library [44]. The poly-CTL-epitope fragment was constructed with the help of genetic algorithm using the PyEvolve library [38]. Biopython library was used to read amino acid sequences written in Fasta or GenBank format [45]. T-helper epitope predictions were based on ProPred models [39]. CTL epitopes predictions were made using our program TEpredict [37].

## Availability and requirements

Project name: PolyCTLDesigner

Project home page: http://polyctldesigner.sourceforge.net

Operating system(s): platform-independent

Programming language: Python

Other requirements: Python 2.7, TEpredict (provided with the PolyCTLDesigner), Biopython, NumPy, PyEvolve, and Python graph

License: Creative Commons Attribution Non-Commercial License V2.0 (CC BY-NC 2.0)

Any restrictions to use by non-academics: see CC BY-NC 2.0 license

## Competing interests

The authors declare that they have no competing interests.

## Authors’ contributions

SIB initiated the studies of polyepitope immunogens at the State Research Center of Virology and Biotechnology “Vector” and has developed the prototype of polyepitope rational design algorithm. DVA completed the concept, chose the models and algorithms, and implemented PolyCTLDesigner. DVA drafted the manuscript. SIB was involved in writing the manuscript and its critical revision and has given the final approval for the version to be published. Both authors read and approved the final manuscript.

## Supplementary Material

Additional file 1 This file describes the short sample study of designing artificial poly-CTL-epitope antigen composed of 6 well studied HIV-1 CTL epitopes using PolyCTLDesigner. Click here for file

Additional file 2 This file contains the source code of PolyCTLDesigner together with a sample workflow script (designer.py). Click here for file
