# Supplementary material for: PolyCTLDesigner: a computational tool for constructing polyepitope T-cell antigens
Source: BMC Res Notes. 2013 Oct 10;6:407. doi: 10.1186/1756-0500-6-407 (PMC3853014; doi:10.1186/1756-0500-6-407)
Supplement: Additional file 1 — This file describes the short sample study of designing artificial poly-CTL-epitope antigen composed of 6 well studied HIV-1 CTL epitopes using PolyCTLDesigner. [file 1756-0500-6-407-S1.zip › SampleStudy.pdf]

Using PolyCTLDesigner to construct the polyepitope antigen from 6 preselected HIV-1 CTL epitopes.

To theoretically illustrate the impact of poly-CTL-epitope optimization we performed the following analysis. From the database of well-defined HIV-1 CTL epitopes (http://www.hiv.lanl.gov/content/immunology/tables/optimal_ctl_summary.htm) we selected 6 HLA-A*02:01 restricted T-cell epitopes: SLYNTVATL, FLGKIWPSYK, VIYQYMDDL, AIIRILQQL, RGPGRAFVTI and VLEWRFDSRL (please, see the file ***selected HIV-1 epitopes.txt*** for more details).

At first we applied our program to rank all 341 spacers derived from degenerate motif [ARLK][LDIT][VLGA][KVAS] for all 30 possible pairs of selected CTL epitopes. Proteasome/immunoproteasome cleavage sites were predicted here with a threshold of 3 top percentiles to increase the specificity of prediction. We ran the following code (from the script ***HIV_6ctls_[ALRK][LDIT][VLGA][KVAS].py***):

```
import os
ctls = ['SLYNTVATL','FLGKIWPSYK','VIYQYMDDL','AIIRILQQL','RGPGRAFVTI','VLEWRFDSRL']
motif = "[['A','L','R','K'],['L','D','I','T'],['V','L','G','A'],['K','V','A','S']]"
for pep1 in ctls:
    for pep2 in ctls:
           if pep1 != pep2:
              ## Here we rank all the spacers for all possible pairs of the epitopes
              ## (10230 combinations = 30 pairs x 341 spacers)
              ## Results are saved into the text file "test_6ctls_all.out"
              os.system('''python epitopes_count.py %s %s "%s" T T 6.3 3 test_6ctls_all.out
PLS_ICA11 all T T T'''%(pep1,pep2,motif))
              ## Here we select the best spacer for each possible pair of epitopes
              ## (30 combinations)
              ## Results are saved into the text file "test_6ctls_best.out"
              os.system('''python epitopes_count.py %s %s "%s" T T 6.3 3 test_6ctls_best.out
PLS_ICA11 all T T F'''%(pep1,pep2,motif))
```

After that we ran the module ***construct_graph.py*** using the following code:

```
> python construct_graph.py test_6ctls_[ALRK][LDIT][VLGA][KVAS]_best_ranked.out
```

Obtained directed weighted graph was saved to file ***test_6ctls_[ALRK][LDIT][VLGA][KVAS]_best_ranked_CTL_graph.dat***.

The graph was analyzed using the script ***DiGraph_analysis.py*** and results (the table with all possible polyepitopes and their weights) were saved to file ***6ctls_[ALRK][LDIT][VLGA][KVAS]_polyE.tab***.

Similar analysis was done using the same peptides and either [A][D][L][V][K][V] or [KR] or [] spacer (in the last case peptides were joined end to end).

It was found that theoretically the spacer choice can have a great impact on proteasomal cleavage efficiency, especially for certain peptides, e.g. for the SLYNTVATL peptide the best spacer had proteasomal processing efficacy of 1 (the top percentile cleavage site), the average efficacy of all tested spacers was 2. The distribution of weights computed with our ranking function had the following parameters: minimal weight was 3.232, the mean weight value was 7.76 and the median value was 7.308. Whereas for peptide FLGKIWPSYK predicted proteasomal cleavage efficacy was the following: the best value was 2, the median was 10 and the average efficacy was 22.6; the distribution of weights had the following parameters: the minimal weight was

6.268, the mean value was 30.542 and the median was 24.081. Thus, theoretically, the choice of spacer sequence does influence the proteasomal processing efficiency, and this effect would be especially prominent for certain peptides.

When all possible polyepitope constructions were tested, it was found that an optimal polyepitope *VIYQYMDDL-ADLK-RGPGRAFVTI-ADGK-SLYNTVATL-ATGS-AIIRILQQL-RDLK-VLEWRFDSRL-ADGK-FLGKIWPSYK* had the weight equal to 18.97, and that only 17 % of all polyepitopic constructs did not contain inefficient proteasomal cleavage sites (those with threshold score below the 3[d] top percentile – inefficient sites were overweighted, their weights were set to 5000) between the target epitopes. It was found that probability of selecting an optimal epitopes permutation at random was less than 0.00139. It's of interest to note that polyepitopes, produced using [A][D][L][V][K][V] spacer motif, demonstrated higher overall weights: the minimal weight was 23.55 (polyepitope *RGPGRAFVTI-ADL-VIYQYMDDL-ADL-VLEWRFDSRL-ADL-SLYNTVATL--AIIRILQQL-ADL-FLGKIWPSYK*), we suppose that it was due to higher numbers of junctional epitopes.

Using the blank spacers (i.e. joining the epitopes end to end) we were unable to find any permutation containing no inefficient cleavage sites between the target epitopes even after lowering the stringency of proteasomal cleavage prediction (with lowering the prediction threshold from the 3[d] top percentile to top decile value, i.e. from 3 to 10) – all polyepitopes contained from 1 to 5 sites with cleavage efficiency below that of the top decile sites. However using only 1-aa long spacer – the [KR] motif – we were able to obtain the polyepitope *VIYQYMDDL--AIIRILQQL-R-SLYNTVATL--RGPGRAFVTI-KVLEWRFDSRL-K-FLGKIWPSYK* with overall weight equal to 50.118.

All the polyepitopes and their weights as well as ranking of all possible spacers for all possible pairs of peptides could be found in file ***polyCTLs.xlsx***.

Thus, theoretically our program allows to select the optimal spacers and to optimize the arrangement of epitopes within the polyepitope. Of course the efficiency of such polyepitope optimization should be thoroughly validated with carefully designed wet-lab experiments. Thus, we would greatly appreciate any critical feedback from our colleagues working in the fields of immunoinformatics, molecular immunology and vaccinology.

In near future we plan to develop GUI for our PolyCTLDesigner program to make it more user friendly.
